# Multifunctional theranostic magnetic PLGA nanoparticles encapsulating cyclosporine A: addressing challenges in pancreas transplantation for type 1 diabetes

**DOI:** 10.3389/fimmu.2026.1746407

**Published:** 2026-02-09

**Authors:** Cátia Vieira Rocha, Andreia Patrícia Magalhães, Lara Diego-González, Victor Gonçalves, Martin Kotrulev, Iria Gomez-Touriño, Manuel Bañobre-López, Juan Gallo

**Affiliations:** 1 International Iberian Nanotechnology Laboratory, Braga, Portugal; 2 Centre for Research in Molecular Medicine and Chronic Diseases (CiMUS), University of Santiago de Compostela, Santiago de Compostela, Spain; 3 Health Research Institute of Santiago de Compostela (IDIS), Santiago de Compostela, Spain

**Keywords:** hypoxia, magnetic nanocomposites, MRI, pancreas transplantation, theranostics, type 1 diabetes

## Abstract

Type 1 Diabetes (T1D) is a high-incidence chronic autoimmune disease, with patients requiring lifelong insulin therapy. In the most severe cases, pancreas transplantation (PTA) arises as the first choice of treatment for these patients in the hope of achieving insulin independence. However, the long-term success of PTA is hindered by ischemia-reperfusion injury (IRI) and immune rejection, both of which limit graft survival. To address these challenges, we have developed multifunctional theranostic nanoparticles (t-PLGA NPs) co-encapsulating Fe_3_O_4_ and MnO NPs, along with cyclosporine A, an immunosuppressive drug. These immunomodulating NPs serve as dual contrast agent for MRI, while generating oxygen to combat hypoxia during IRI. The t-PLGA NPs exhibit efficient drug encapsulation and sustained release, enhancing immunosuppression while minimizing systemic toxicity. *In vitro* studies also demonstrated the NPs’ ability to suppress the immune system, validating the NPs’ potential to prevent graft rejection. The combination of imaging and therapeutic properties makes this platform highly promising for improving PTA outcomes in T1D patients.

## 1 Introduction

Diabetes Mellitus is one of the most prevalent and rapidly increasing health challenges worldwide, with the affected population being predicted to rise from 527 million to 784 million by 2045 (1). Among those affected, almost 9 million currently suffer from type-1 diabetes (T1D), an autoimmune disease that typically requires lifelong insulin therapy. Though widely used, insulin therapy requires regular injections or continuous subcutaneous infusions via insulin pumps, which disrupt patient’s daily life (1, 2).

Pancreas transplantation (PTA) is a vital therapeutic option for patients with severe T1D, particularly those who suffer from frequent hypoglycemia unawareness and metabolic complications that are unmanageable with insulin therapy alone, or those with T1D and end-stage renal disease. While PTA offers the potential to restore endogenous insulin production and stabilize blood glucose levels, this procedure comes with its own significant risks. Despite high early survival rates of 96-98% in the first year and 90% at five years (3), long-term success is often hampered by ischemia-reperfusion injury (IRI) and immune rejection (4) (5),. These complications represent major obstacles to improving the outcomes of PTA, emphasizing the urgent need for new approaches to protect the graft and enhance patient outcomes.

IRI can be divided into two phases: the initial ischemic phase, with cellular damage resulting from the triggering of inflammatory responses due to oxygen deprivation and ATP depletion from the lack of blood flow; and the reperfusion phase, where the sudden restoration of blood flow generates an eruption of reactive oxygen species (ROS), which further exacerbates cellular damage by triggering mitochondrial dysfunction and immune activation (5–8). This cascade of events leads to inflammation, immune responses and, ultimately, graft rejection. Since IRI is considered the main cause of early graft dysfunction and an important risk factor for late-stage allograft failure, new solutions are needed to help mitigate this event (5, 9). Additionally, the long-term use of systemic immunosuppressants, though essential for preventing immune rejection, can result in serious side effects (3, 4). To address these challenges, here we propose a comprehensive approach that integrates cutting-edge nanotechnology and controlled immunosuppression to enhance graft survival, while mitigating the effects of IRI.

Advanced nanotechnology strategies can be employed for both the treatment and monitoring of transplanted organs. These approaches can help target the effect of immunosuppressive medications, allowing a reduction in side effects while generating oxygen *in situ* to mitigate hypoxic conditions resulting from limited blood flow. Additionally, they offer means for longitudinal treatment monitoring through medical imaging technologies. Herein, we offer the use of theranostic multifunctional nanoparticles (NPs) composed of poly(lactic-co-glycolic acid) -PLGA-, a biocompatible and eco-friendly copolymer approved by both the European and American medicine agencies (EMA and FDA) (10, 11),. PLGA is a well-established material in nanotechnology, capable of encapsulating a wide range of substances, including small drugs (10, 12, 13) vaccines (14), proteins (15, 16) and metallic (17) or magnetic nanoparticles (MNPs) (18, 19). The selection of PLGA is not only due to its low toxicity, but also its versatility that allows the adjustment during synthesis of parameters such as size, hydrophobicity and degradation rate (20).

Cyclosporine A (cycA) is a selective immunosuppressant with anti-inflammatory properties, widely used to prevent transplant rejection and autoimmune T cell-mediated diseases (20, 21). CycA binds to cyclophilin forming a complex that inhibits calcineurin, a protein phosphatase involved in the activation of T-lymphocytes. In this way, the nuclear factor of activated T cells (NFAT) is blocked, suppressing the transcription of interleukin-2 (IL-2) which, subsequently, inhibits the activation of T cells and their differentiation (22, 23). Although less pronounced, the effect of cycA extends to other immune cells contributing to its overall immunosuppressive effect (24). CycA also inhibits the mitochondrial permeability transition pore. By blocking the pore opening, cycA helps prevent cell death in transplanted tissues (25). Since CycA can cause significant off-target effects (26), its encapsulation is essential for safe immunosuppression.

Magnetic nanoparticles (MNPs) are being extensively studied for biomedical applications due to their magnetic properties, which allow them to respond to external magnetic fields, and their proven biocompatibility. Furthermore, some MNPs are sensitive to changes in pH and redox conditions. This is the case of MnO NPs. These nanoparticles, due to their paramagnetic nature, that is, they do not retain magnetization in the absence of an external magnetic field (27, 28) have been widely proposed as *T_1_* contrast agents (CAs) in MRI for medical imaging and can also help mitigate hypoxic conditions through *in situ* oxygen generation (13). Manganese oxides have also been reported as ROS scavengers, due to their catalytic antioxidant properties (29, 30). Iron oxide NPs are broadly used in nanomedicine, displaying superparamagnetic behavior when their size is below a critical diameter (<15 nm) (31). This property allows them to be utilized in theranostics, combining magnetic hyperthermia and *T_2_*-weighted MR imaging. The unique characteristics of MNPs have prompted extensive research into their encapsulation within nanocomposites, aiming to combine their outstanding functional properties with, for example, the drug delivery capacity of organic systems, and thus obtain structures with theranostic capabilities (32).

**Figure 1:**
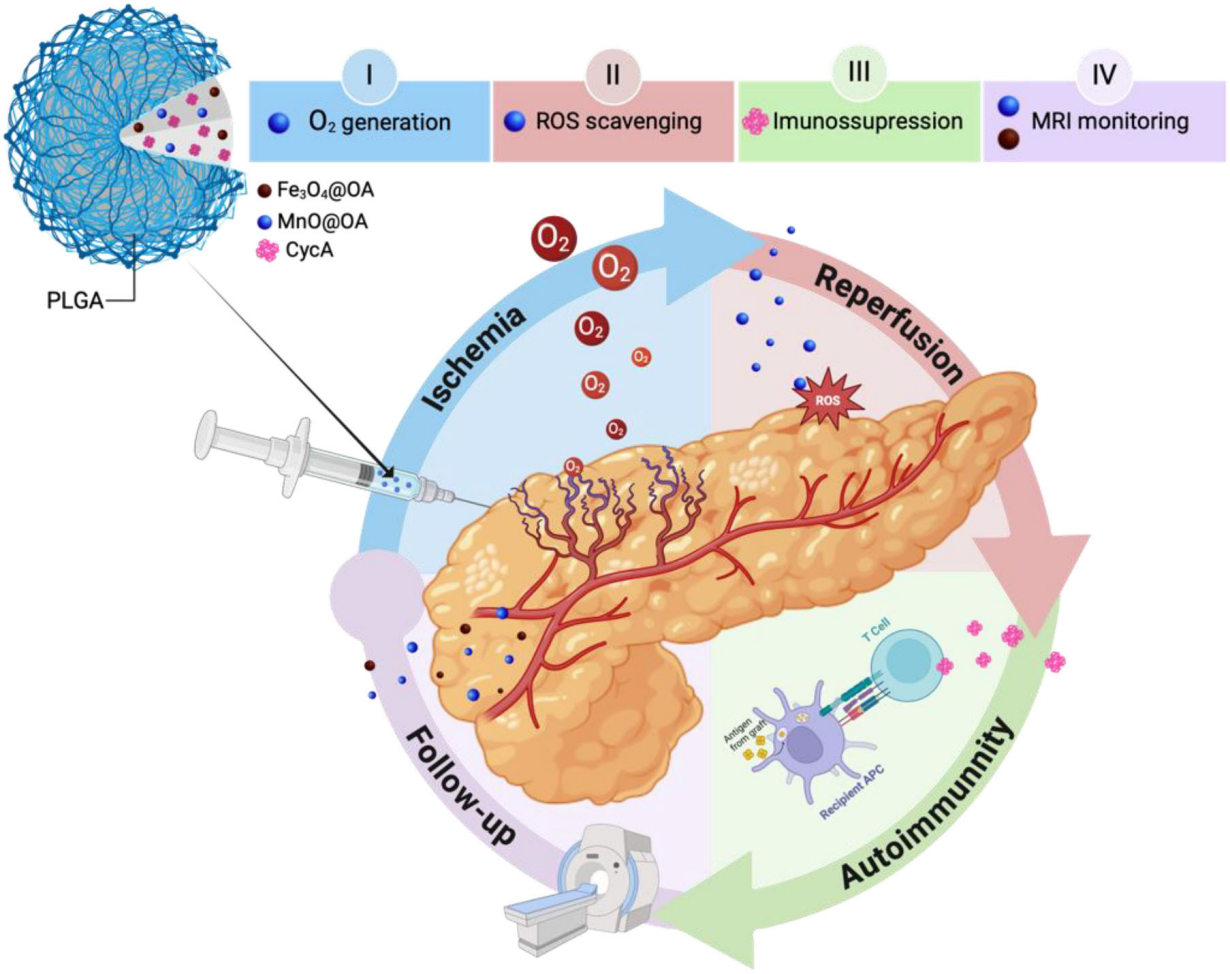
Schematic representation of the proposed holistic mechanism of action of tPLGA-NPs in the different phases of PTA.

The integration of nanotechnology and immunosuppression offers a novel approach to transplantation medicine. This study proposes the utilization of theranostic PLGA NPs (t-PLGA NPs) to mitigate the challenges associated with pancreas transplantation (PTA), including hypoxia, oxidative stress-induced cell death, allograft rejection, and the adverse effects of immunosuppression, while simultaneously facilitating longitudinal graft monitoring via non-invasive MRI (**Figure 1**). The (para)magnetic properties of the proposed system, enable its use as a dual *T_1_-T_2_* MRI contrast agent, providing real-time monitoring of the transplant. Beyond imaging, the NPs act as an O_2_ generator, helping restore O_2_ levels to counter reduced blood flow effects during IRI. Additionally, the NPs serve as a controlled drug delivery platform to suppress immune and inflammatory responses, while simultaneously scavenging ROS to prevent mitochondrial damage and cell death, especially during the critical reperfusion phase. The proposed nanosystem represents an unprecedented convergence of (para)magnetic properties, oxygen-generating capacity, immunosuppressive drug delivery, and reactive oxygen species scavenging activity within a unified platform—a combination that has not been previously documented in transplantation research. This innovative strategy is designed to improve the overall outcome of pancreas transplantation by preserving pancreatic function and reducing the incidence of graft rejection by preventing the deleterious effects of IRI. This approach offers renewed hope for patients with T1D, having the potential to enhance both the efficacy and durability of PTA.

## 2 Materials and methods

### 2.1 Chemicals

Resomer^®^ RG 752 H, Poly(D,L-lactide-*co*-glycolide) copolymer, monomer ratio 75:25, Mw=4000–15000 Da, Poly(vinyl alcohol) (PVA), Mw=9000-10000, 80% hydrolyzed, dichloromethane (DCM), acetonitrile HPLC grade (>99.93%), trifluoroacetic acid (TFA) HPLC grade (≥99%), Phosphate-buffered saline (PBS), hydrogen peroxide solution (30% w/w), and Tris(2,2′-bipyridyl)dichlororuthenium(II) hexahydrate were purchased from Merck KGaA (Darmstadt, Germany). Cyclosporine A was obtained from ADOOQ Bioscience (CA, USA). DiO’ (3,3’-Dioctadecyloxacarbocyanine Perchlorate) was purchased from ThermoFisher Scientific (Dreieich, Germany).

### 2.2 Methods

#### 2.2.1 Synthesis and characterization of theranostic PLGA nanoparticles

Fe_3_O_4_@OA nanoparticles were synthesized using a co-precipitation method previously outlined by Almeida et al. (33), while the preparation of MnO@OA was carried out through the thermal decomposition of MnOA_2_ as described in ref (34).

The synthesis and characterization of the theranostic capabilities *in vitro* of PLGA NPs followed the exact protocols described in ref (35).

#### 2.2.2 Evaluation of CycA loading and release kinetics

To evaluate CycA loading efficiency, the supernatants collected after NP purification were analyzed to quantify the non-encapsulated CycA using reverse-phase high-performance liquid chromatography (RP-HPLC; Agilent 1290 Infinity II LC System). Chromatographic separation was carried out at 70 °C using a C18 column (15 cm × 0.21 cm, 5 µm; Teknokroma TR-025078), applying a gradient method with acetonitrile and water containing 0.1% TFA) as the mobile phase at a flow rate of 0.5 mL/min, and the following protocol was used: starting from 70:30, get to 25:75 in 10 min; 25:75 for 2 min; to 10:90 in 3 min; back to 70:30 in 0.1 min; and 70:30 for 3 min. Detection was performed at 314 nm.

The encapsulation efficiency (EE%) was calculated as:


$$EE\%=\frac{\left[\text{encapsulated drug}\right]}{\left[\text{total drug}\right]}\;\times 100$$

To assess drug release kinetics, 0.6 mL of the nanoformulation was loaded into a 6 kDa Pur-A-Lyzer dialysis device (Merck KGaA) and submerged in 10 mL of PBS:ethanol (9:1). The system was incubated at 37 °C for 14 days, with 0.5 mL aliquots collected at predetermined time points for HPLC quantification of cumulative CycA release.

#### 2.2.3 Biological assays

This study was approved by the Comité de Ética de la Investigación de Santiago-Lugo, Xunta de Galicia (2021/412). Informed consent was obtained from the study participants, and the guidelines outlined in the Declaration of Helsinki were followed.

Human PBMCs were isolated from whole blood by Ficoll-based density centrifugation (GE HealthCare) and maintained in RPMI (Merck KGaA) supplemented with 10% heat-inactivated FBS and 1% penicillin/streptomycin at 37 °C in 5% CO_2_ atmosphere. RIN-m cells (ATCC-CRL2057) were cultured under the same conditions, with an additional 1% non-essential amino acids. Passaging occurred every 3–4 days at 70–80% confluency. For immunosuppression assays, human AB serum (Merck KGaA) (10%) replaced FBS.

#### 2.2.4 Biocompatibility evaluation

AquaBluer (MultiTarget Pharmaceuticals LLC) was used to assess cell viability after 48 h exposure to NPs (CycA: 0.1–50 µg/mL). PBMCs (5 × 10^4^ cells/well) and RIN-m (1 × 10^4^ cells/well) were seeded into 96-well plates (TPP™) and pre-incubated for 24 h at 37 °C and 5% CO_2_ before treatment. Afterwards, the nanoparticles were applied and incubated for 48 hours. Post-treatment, AquaBluer (1:100 dilution) was added and incubated for 4 h. Fluorescence (λ_ex_ = 540 nm, λ_em_ = 590 nm) was recorded on a SYNERGY H1 reader (Biotek). Cell viability was calculated following the manufacturer instructions.

#### 2.2.5 Immunosuppression assay

PBMCs were seeded in 96-well round-bottom plates at a density of 2 × 10^5^ cells/well. Cells were stimulated with phorbol 12-myristate 13-acetate and ionomycin (PMA/I) at final concentrations of 0.02 and 1 µg/mL, respectively. Following a 24 h incubation period, the cells were centrifuged (500 g, 10 min) and the supernatants discarded. Meanwhile, free CycA and tPLGA NPs were diluted in RPMI media at CycA concentrations of 0.4, 0.8 and 1 µg/mL. Unstimulated cells were used as the negative control, while cells stimulated with PMA/IO served as the positive control. The cells were then treated with 200 µL of either free CycA or tPLGA-NPs and incubated for an additional 24 h. The following day, the cells were centrifuged (500 g, 10 min), the supernatants were collected and stored at -80 °C for subsequent analysis. Cytokines (IL-2 and IFN-**γ)** were measured in each supernatant. The concentration of each cytokine was measured using specific ELISA kits (ELISA MAX™) according to the manufacturer’s instructions (BioLegend, San Diego, CA, USA).

#### 2.2.6 Flow cytometry

For flow cytometry studies PBMCs from healthy donors were seeded in 96-well round-bottom plates at a density of 2 × 10^5^ cells/well. Cells were stimulated with Phorbol 12-Myristate 13-Acetate (PMA) and Ionomycin (I) at final concentrations of 0.02 and 1 µg/mL, respectively. Following a 24 h incubation period, the cells were centrifuged (500 g, 10 min) and the supernatants discarded. Meanwhile, free CycA and tPLGA NPs were diluted in RPMI media at CycA concentrations of 0.4, 0.8 and 1 µg/mL and the cells were then treated with 200 µL of either free CycA or tPLGA-NPs and incubated for additional 24 h. Later, PBMCs were transferred to 96-well V-bottom plates and washed with DPBS. Next the cells were incubated with Aqua at a dilution 1:1000 following the manufacturer’s instructions for live/dead cell quantification. The cells were then washed, acquired using a Cytoflex S (Beckman Coulter, CA, USA) and the data analysis was performed using the CytExpert software (V2.4, Beckman Coulter).

## 3 Results and discussion

### 3.1 Synthesis and physicochemical characterization of tPLGA NPs

**Figure 2:**
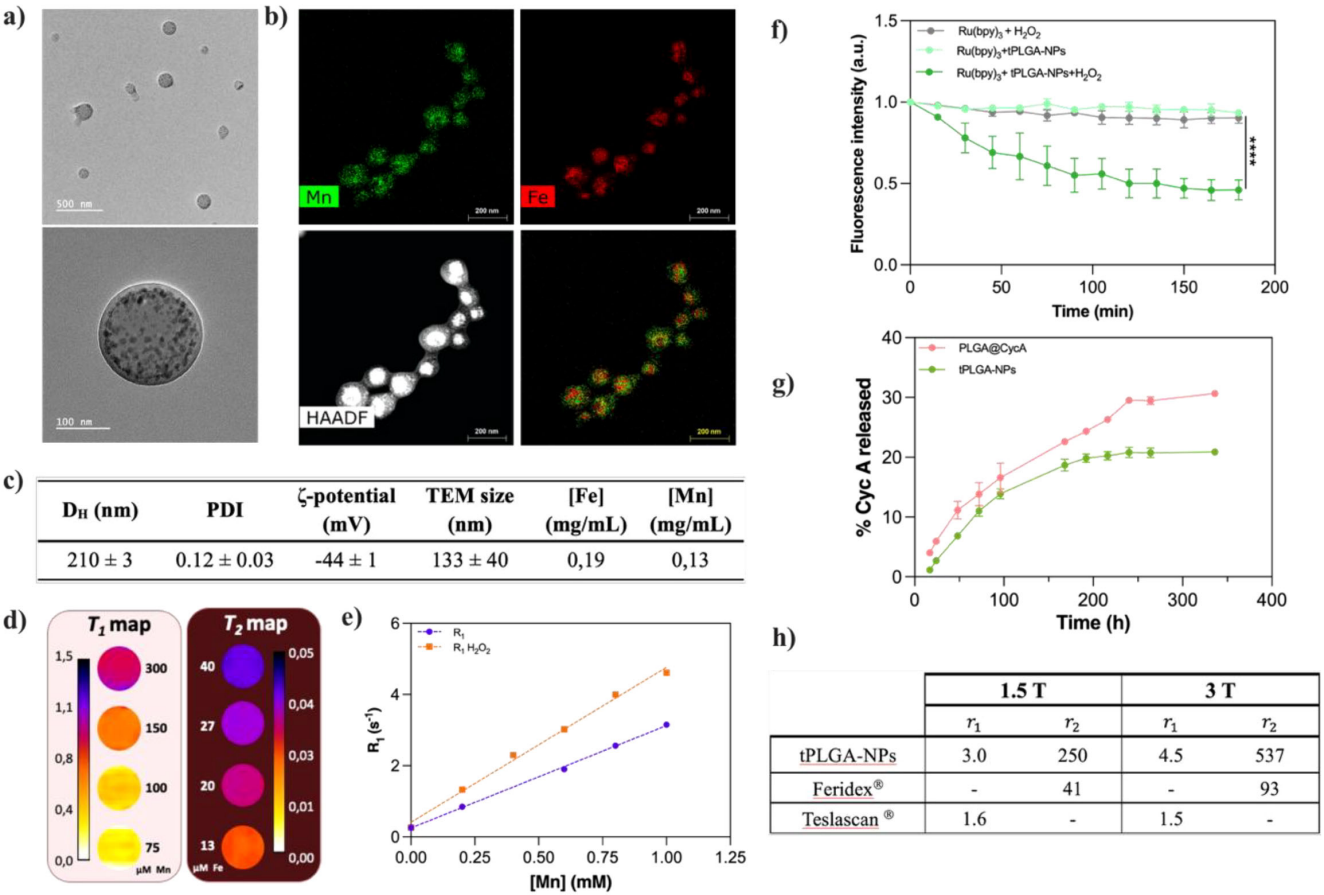
**(a)** TEM images of tPLGA NPs and **(b)** respective elemental mapping by STEM-EDX; **(c)** Physico-chemical characterization of the tPLGA NPs by DLS, TEM and ICP; **(d)** Pseudo-colored MRI parametric maps of tPLGA NPs serial dilutions, left: T_1_ map; right, T_2_ map; **(e)** relaxometry measurements of PLGA@MnO NPs in the presence and absence of H_2_O_2_ as oxidizing agent; **(f)** Evolution of the emission intensity of Ru(bpy)_3_ along a 3 h period (grey – control with Ru(bpy)_3_ and oxidizing agent; light green – control with Ru(bpy)_3_ and tPLGA- NPs; dark green – sample with Ru(bpy)_3_, tPLGA- NPs and oxidizing agent); **(g)** Drug release profile of tPLGA-NPs (green) and control formulation without magnetic nanoparticles but with CycA (pink); and **(h)** Table summarizing the longitudinal (r_1_) and transversal (r_2_) relaxivities of the tPLGA NPs and commercially available Mn- and Fe-based formulations, measured at 1.5 and 3.0 T (36).

Multifunctional theranostic nanoparticles (tPLGA NPs) were synthesized using a reproducible double emulsion (W/O/W) method (32), designed to co-encapsulate both hydrophilic and hydrophobic components. TEM revealed the successful formation of spherical nanostructures with an average diameter of 133 ± 40 nm (**Figure 2a**). Within the PLGA matrix, the successful encapsulation of high-density inorganic nanoparticles was visible as darker, hypointense regions. To confirm the co-encapsulation and distribution of the magnetic components, STEM-EDX mapping was performed. Elemental maps clearly verified the presence of both iron (Fe) and manganese (Mn) within individual PLGA nanoparticles (**Figure 2b**). Interestingly, the mapping suggested a degree of spatial heterogeneity, with Fe-based NPs tending to cluster towards the core and Mn-based NPs localizing more peripherally.

Dynamic light scattering (DLS) analysis indicated a hydrodynamic diameter (D_h_) of 210 ± 3 nm with a low polydispersity index (PDI) of 0.12 ± 0.03, confirming a monodisperse population in aqueous solution (**Figure 2c**). The larger size observed by DLS compared to TEM is expected, as DLS measures the hydrodynamic diameter, which includes the polymer corona and associated hydration shell, while TEM visualizes the dehydrated state (37). The nanoparticles exhibited a significant negative surface charge (ζ-potential) of -44 ± 1 mV, which is indicative of high colloidal stability and is crucial for preventing aggregation upon systemic administration. The successful incorporation of all components was further validated by FTIR (**Supplementary Figure S1**). The spectrum of the final tPLGA NPs was dominated by the characteristic peaks of the PLGA polymer, including C-H stretching vibrations below 3000 cm^-1^, a strong carbonyl (C=O) stretch at approximately 1700 cm^-1^ and C-O stretching bands between 1000–1300 cm^-1^. The presence of CycA was confirmed by the persistence of its characteristic C-H bending vibrations around 1500 cm^-1^. Furthermore, a subtle peak around 550 cm^-1^, attributable to the metal-oxygen bonds of the iron and manganese oxides, confirmed the successful loading of the inorganic nanocrystals within the final composite structure.

### 3.2 Optimization of magnetic properties for dual-mode MRI

A primary objective of this work was to engineer a nanoparticle system capable of acting as a dual-mode *T_1_-T_2_* contrast agent for non-invasive MRI monitoring. This required careful optimization of the magnetic payload. The magnetic properties of the tPLGA NPs, base of their MR performance, were investigated using magnetometry, which revealed a mixed superparamagnetic-paramagnetic profile, resulting from the combination of superparamagnetic Fe_3_O_4_ and paramagnetic MnO NPs, characterized by the absence of hysteresis and S-shaped magnetization curves without saturation (**Supplementary Figure S2**).

To achieve an optimal balance for dual-mode MR imaging, a systematic study was conducted by varying the Fe: Mn ratio (**Table 1**). While a higher Mn content (e.g., 1:2 Fe: Mn ratio) was found to be ideal for maximizing *T_1_* contrast, this led to significant synthetic challenges and a lower overall metal loading. Consequently, a 1:1 Fe: Mn ratio was selected as a pragmatic compromise, ensuring sufficient concentrations of both metals for effective *T_1_-T_2_* contrast while maintaining manufacturability. The relaxometric properties of the optimized tPLGA NPs were evaluated at clinical field strengths of 1.5 T and 3.0 T (**Figures 2d, h**). The nanoparticles demonstrated excellent performance as a *T_2_* contrast agent (13, 38) with a transverse relaxivity of 250 mM^-1^s^-1^ at 1.5 T and 537 mM^-1^s^-1^ at 3.0 T. These values are substantially higher than those of the commercial iron-based agent Feridex^®^ (*r_2_* = 41 and 93 mM^-1^s^-1^ at 1.5 T and 3.0 T, respectively). Concurrently, the NPs exhibited strong *T_1_* contrast, with a longitudinal relaxivity of 3.0 mM^-1^s^-1^ at 1.5 T and 4.5 mM^-1^s^-1^ at 3.0 T (**Figure 2d**), surpassing the manganese-based agent Teslascan^®^ (*r_1_* = 1.6 and 1.5 mM^-1^s^-1^, **Figure 2h**) (36). The unexpected increase in *r_1_* at the higher field strength may arise from the complex interplay and coupling effects between the *T_1_* and *T_2_* moieties within the nanocomposite, a phenomenon previously observed in intricate dual-mode systems (39).

**Table 1:** Relaxivity values of the samples prepared with different Fe, Mn ratios.

Ratio Fe: Mn	MRI			Final [Fe] mg/mL	Final [Mn] mg/mL
	r_1_	r_2_	r_2/_r_1_		
1:0.2	–	421	–	0.31	0.07
1:0.3	–	407	–	0.34	0.10
1:0.4	–	419	–	0.31	0.13
1:0.5	–	468	–	0.31	0.16
1:0.6	0.6	504	840	0.29	0.17
1:0.7	3.3	251	76	0.19	0.13
1:2	1.7	106	61.7	0.04	0.08
1:4	1.7	106	98.2	0.02	0.06

### 3.3 Therapeutic functionality: oxygen generation and controlled immunosuppression

A key therapeutic strategy of this platform is to mitigate IRI by generating oxygen *in situ*. The ability of the MnO component to produce O_2_ in a redox-active environment was first assessed using a Ru(bpy)_3_-based fluorescence quenching assay (**Figure 2f**). In the presence of H_2_O_2_, a significant, time-dependent decrease in fluorescence intensity was observed exclusively for the sample containing tPLGA NPs, confirming catalytic O_2_ generation (30, 40, 41). This was further corroborated by relaxometry, where the *r_1_* value of MnO-containing NPs increased from 2.8 to 4.4 mM^-1^s^-1^ in the presence of H_2_O_2_, consistent with the reduction of MnO and release of paramagnetic Mn²^+^ ions that accompanies oxygen production (30) (**Figure 2e**).

The second therapeutic function is the controlled delivery of the immunosuppressant CycA. The encapsulation of the hydrophobic drug was highly efficient (>85%), even with the co-loading of inorganic MNPs. The choice of PLGA with a 75:25 lactic-to-glycolic acid ratio was intended to slow polymer degradation and prolong drug release (10, 32). Release kinetics studies over 15 days showed a sustained release profile, with the tPLGA NPs releasing approximately 20% of their payload, compared to 30% for control PLGA NPs lacking MNPs (**Figure 2g**). This suggests the MNP-loaded matrix creates a more tortuous path for drug diffusion and polymer degradation, enabling extended release (18, 32, 42). Kinetic modeling of the release data showed the best fit with the Korsmeyer-Peppas model (K_m_= 0.882 ± 0.215; n=0.575 ± 0.052; AIC = 46.83; R²=0.902), yielding a release exponent (n) of 0.575. This value, falling between 0.5 and 1, indicates an anomalous, non-Fickian release mechanism driven by a combination of drug diffusion and polymer matrix erosion (43), which is ideal for long-term, controlled immunosuppression.

### 3.4 Biocompatibility and cellular interactions

**Figure 3:**
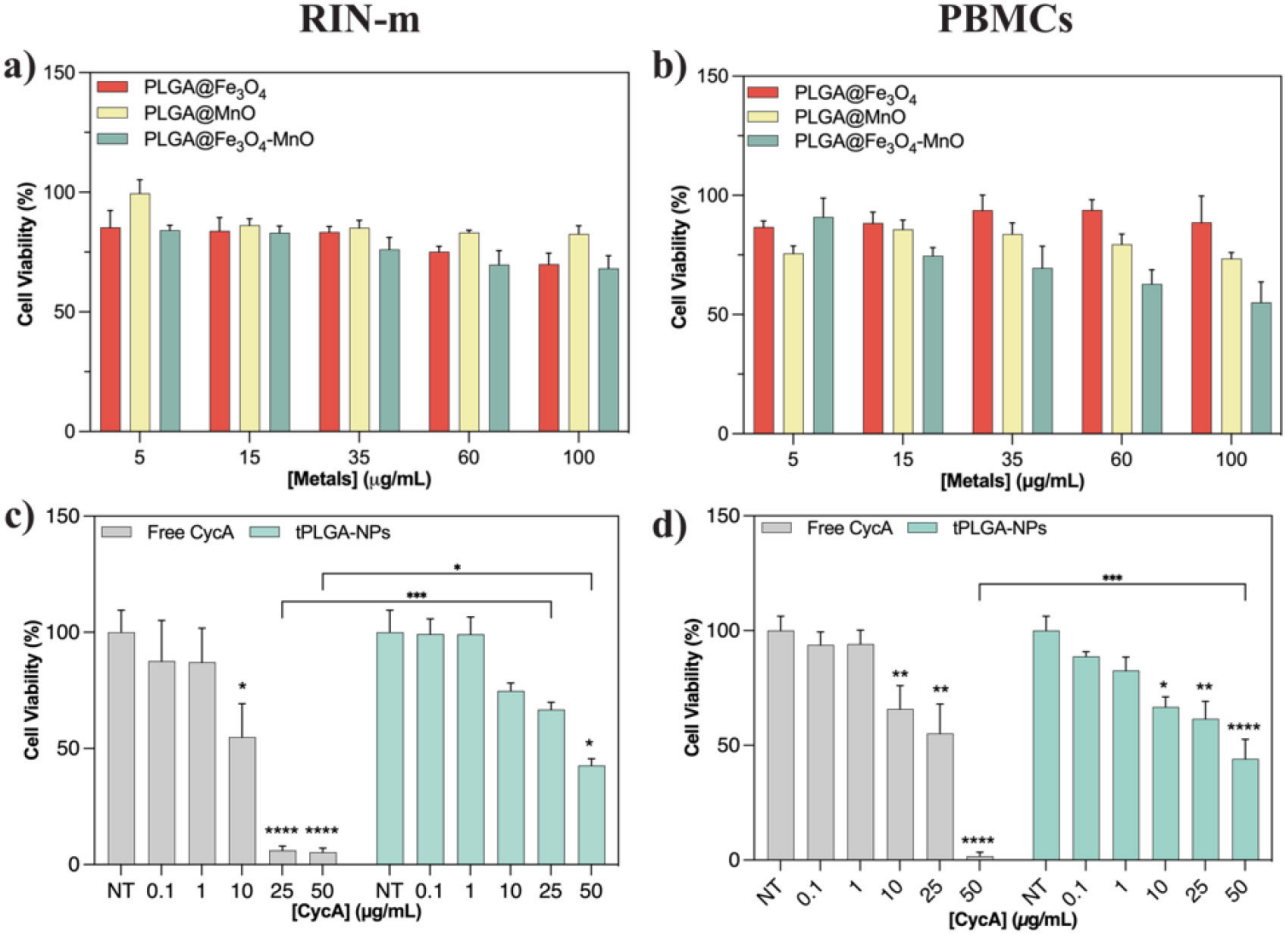
Effect of control formulations on the cell viability of RIN-m cells **(a)** and PBMCs **(b)**. Effect of free cycA and tPLGA-NPs on the cell viability of RIN-m cells **(c)** and PBMCs **(d)**. The data represents the mean value and SEM from a minimum of three independent experiments (NT – non treated; *p<0.05, **p<0.005, ***p<0.0005, ****p<0.0001).

The clinical translation of any nanomedicine hinges on its safety. The cytocompatibility of the tPLGA NPs was evaluated in both rat pancreatic β-cells (RIN-m) and human peripheral blood mononuclear cells (PBMCs). Control formulations without CycA showed no significant cytotoxicity at metal concentrations up to 15 µg/mL (**Figures 3a, b**), a dose well above that required for imaging or therapeutic effects. This confirms the safety of the nanoparticle vehicle itself. Subsequently, the cytotoxicity of free CycA was compared to that of the encapsulated drug (**Figures 3c, d**). In both cell lines, free CycA induced significant dose-dependent toxicity. In contrast, the tPLGA NPs demonstrated a markedly improved safety profile, with significant toxicity only appearing at the highest concentration tested (50 µg/mL). This crucial result highlights a major advantage of nanoencapsulation: the ability to shield cells from the off-target toxicity of the free drug, thereby widening the therapeutic window.

**Figure 4:**
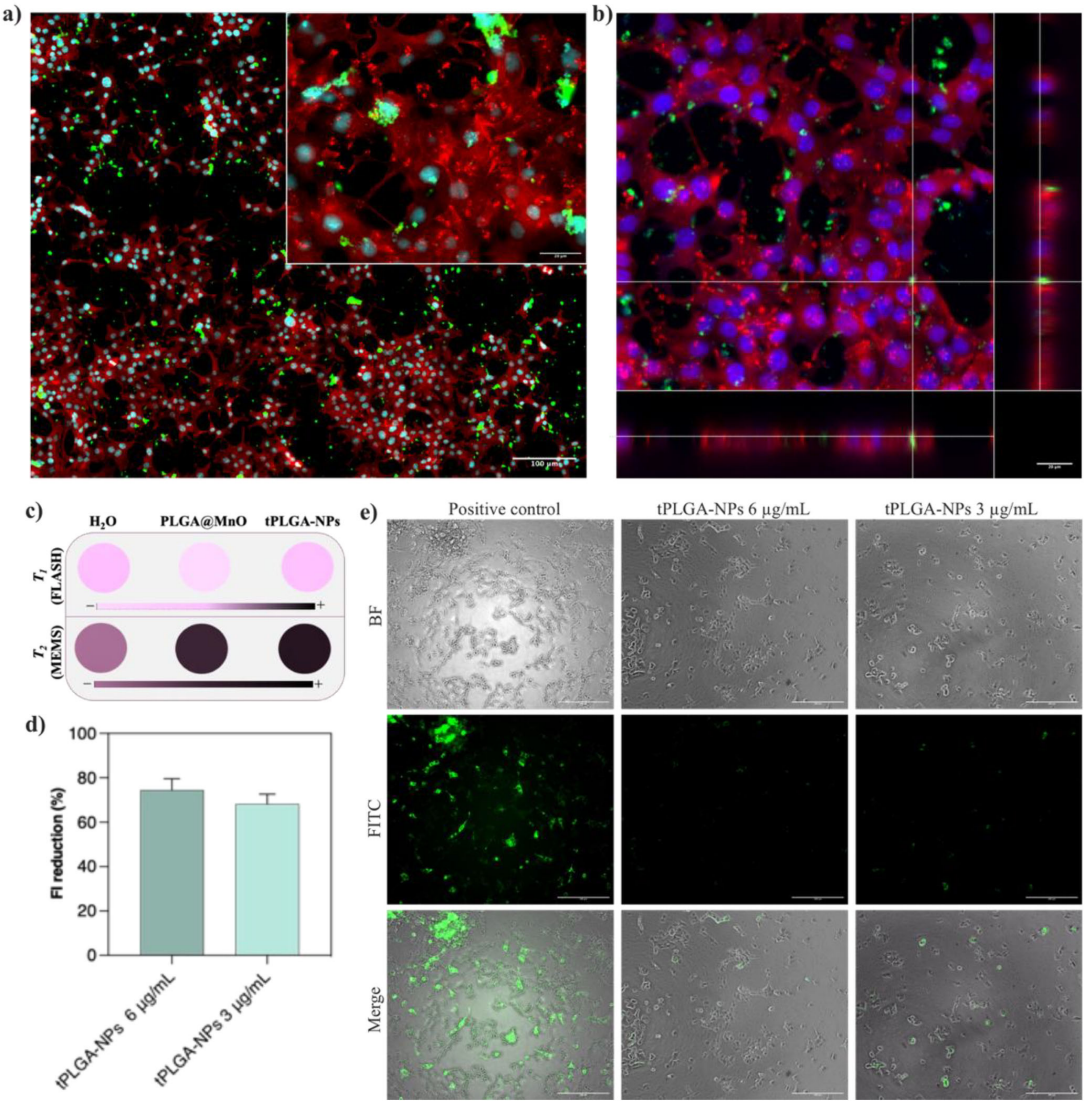
**(a)** Fluorescence microscopy and **(b)** confocal microscopy images of RIN-m cells incubated with 15 μg/mL; **(c)** MRI parametric maps of PLGA@MnO NPs and tPLGA-NPs; top: T_1_ map, bottom, T_2_ map; **(d)** Quantification of the hypoxia remediation capacity of tPLGA NPs in RIN-m cells at two different Mn doses; and **(e)** representative fluorescence microscopy images of RIN-m cell labelled for hypoxia and treated with the tPLGA NPs at two Mn doses (3 and 6 mg Mn/mL). of tPLGA-NPs (metal content) for 24 (h) The nuclei are stained with DAPI, the cytoplasm with α-tubulin and t-PLGA NPs with DiO (λex/em= 489/506 nm), being visible in the blue, red and green channel, respectively.

Effective therapeutic action requires nanoparticle internalization. Cellular uptake was confirmed using confocal and fluorescence microscopy after incubating RIN-m cells with DiO-labeled tPLGA NPs. The images revealed efficient and homogenous uptake, with nanoparticles predominantly localizing in the cytoplasm (**Figures 4a, b**). This confirms that the NPs can effectively enter target cells to deliver their therapeutic payload and to serve as intracellular labels for MRI monitoring.

### 3.5 Validation of theranostic capabilities *in vitro*

Having established the individual functionalities, the integrated theranostic performance of tPLGA NPs was evaluated in relevant cell models. RIN-m cells incubated with tPLGA NPs (containing 2.2 µg/mL Mn and 2.9 µg/mL Fe) generated significant MRI contrast. A notable *T_2_* shortening effect (*T_2_* = 184 ms vs 936 ms for water) and a detectable *T_1_* effect (*T_1_* = 2,291 ms vs 2,387 ms for control) were observed, confirming the potential for *in vitro* cell tracking (**Figure 4c**). The capacity of the tPLGA NPs to remediate hypoxic conditions was then assessed in RIN-m cells. Using a hypoxia-sensitive fluorescent kit, a significant dose-dependent reduction in fluorescence in cells treated with tPLGA NPs was observed, indicating successful O_2_ generation and alleviation of hypoxia (**Figures 4d, e**).

**Figure 5:**
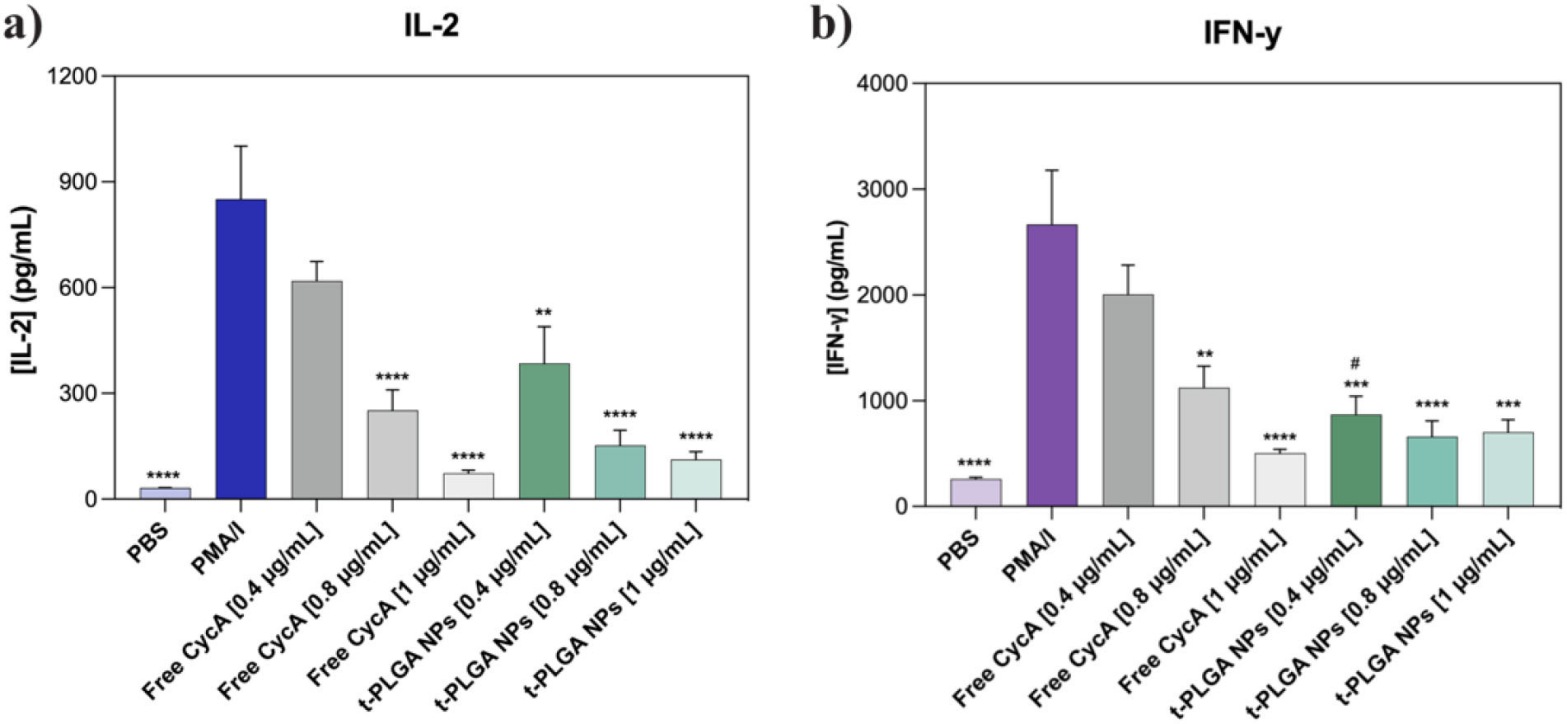
**(a)** Effect of different free CycA concentrations and tPLGA-NPs on activated PBMCs on IL-2 secretion and **(b)** IFN-γ production. *Represent a comparison with positive control and # represents a comparison with free CycA at the same concentration (*p<0.05, **p<0.005, ***p<0.0005, ****p<0.0001; #p<0.05).

Finally, the immunosuppressive efficacy of the formulation was tested. Activated PBMCs treated with tPLGA NPs showed a profound, dose-dependent reduction in the secretion of both IL-2, a key cytokine for T-cell proliferation, and IFN-γ, a major pro-inflammatory cytokine (**Figures 5a, b**) (44). Critically, at equivalent CycA concentrations, the tPLGA NPs demonstrated a significantly greater suppressive effect than the free drug. For instance, at a concentration of 0.4 µg/mL, the tPLGA NPs markedly reduced cytokine levels, whereas free CycA had a negligible effect. This enhanced efficacy, achieved at non-toxic concentrations (**Supplementary Figures S3**, **S4**), highlights the benefit of the nanoparticle delivery system, which can potentiate the drug effect and suppress key pathways of allograft rejection (45, 46).

### 3.6 t-PLGA NPs administration protocol

The proposed t-PLGA NP formulation is designed for initial *ex vivo* application, ensuring that the primary drug release occurs locally at the pancreatic graft site, thereby establishing a robust protective microenvironment prior to transplantation. Subsequent administrations will be delivered systemically, necessitating careful consideration of targeting specificity. In the context of transplantation, an optimal balance must be achieved between local and systemic immunosuppression; while systemic approaches more comprehensively address the multifactorial mechanisms underlying graft rejection, localized immunosuppression minimizes systemic exposure to potent immunosuppressive agents and their associated toxicities. Targeting specificity may be enhanced through exploitation of the magnetic properties of the nanoparticles via magnetic guidance systems, or through incorporation of targeting ligands in future iterations of the platform. Regardless of the approach employed, comprehensive *in vivo* preclinical studies will be required to establish the optimal dosing regimen that maximizes therapeutic efficacy while minimizing toxicity in this clinical setting.

## 4 Conclusion

The tPLGA NPs developed in this study demonstrated appropriate characteristics for systemic administration, including optimal size, charge and low polydispersity, while exhibiting a combination of magnetic behaviors, suitable for dual imaging applications. tPLGA-NPs presented both longitudinal and transversal MRI contrast and their ability to generate oxygen, attributed to the inclusion of MnO, is essential for mitigating hypoxia during the ischemia and reperfusion phases. Additionally, the nanocarriers were effectively engineered to encapsulate CycA, enabling a controlled and sustained drug release, which proved successful in achieving therapeutic goals. This controlled release is essential for sustaining optimal immunosuppression, protecting the transplanted organ from rejection while minimizing systemic side effects and cellular toxicity. Moreover, the particles demonstrated excellent biocompatibility, showing no significant toxicity in donor PBMCs or rat β-cells at therapeutic relevant concentrations, and were efficiently internalized by the latter. tPLGA-NPs also exhibited remarkable MRI contrast generation and effective hypoxia mitigation *in vitro* in insulin-producing cells, even at minimal metal concentrations. ELISA assays demonstrated that the NPs efficiently contribute to T-cell deactivation, highlighting their efficacy in suppressing the immune system. Additionally, the NPs promote a general anti-inflammatory profile, by suppressing the release of pro-inflammatory cytokine (IFN-γ), further enhancing their potential to reduce immune-mediated damage and improve graft survival.

The synergic multimodal abilities of the proposed tPLGA NPs demonstrate their potential to improve transplantation success rates, offering a promising platform for targeted immunosuppressive therapy.

## Data Availability

The original contributions presented in the study are included in the article/**Supplementary Material**. Further inquiries can be directed to the corresponding author.
