# Supplementary material for: Multifunctional theranostic magnetic PLGA nanoparticles encapsulating cyclosporine A: addressing challenges in pancreas transplantation for type 1 diabetes
Source: Front Immunol. 2026 Feb 9;17:1746407. doi: 10.3389/fimmu.2026.1746407 (PMC12926096; doi:10.3389/fimmu.2026.1746407)
Supplement: Supplementary file 1 [file DataSheet1.pdf]

Multifunctional theranostic magnetic PLGA nanoparticles encapsulating cyclosporine A: addressing challenges in pancreas transplantation for type 1 diabetes.

Cátia Vieira Rocha[1,2], Andreia Patrícia Magalhães[1], Victor Gonçalves[1], Lara Diego-González[1], Martin Kotrulev[2,3], Iria Gomez-Touriño[2,3], Manuel Bañobre-López[1], Juan Gallo[1]

[1]International Iberian Nanotechnology Laboratory, Braga, Portugal;
[2]Centre for Research in Molecular Medicine and Chronic Diseases (CiMUS), University of Santiago de Compostela, Santiago de Compostela, Spain.
[3]Health Research Institute of Santiago de Compostela (IDIS), Santiago de Compostela, Spain.

Support information

Magnetic characterization

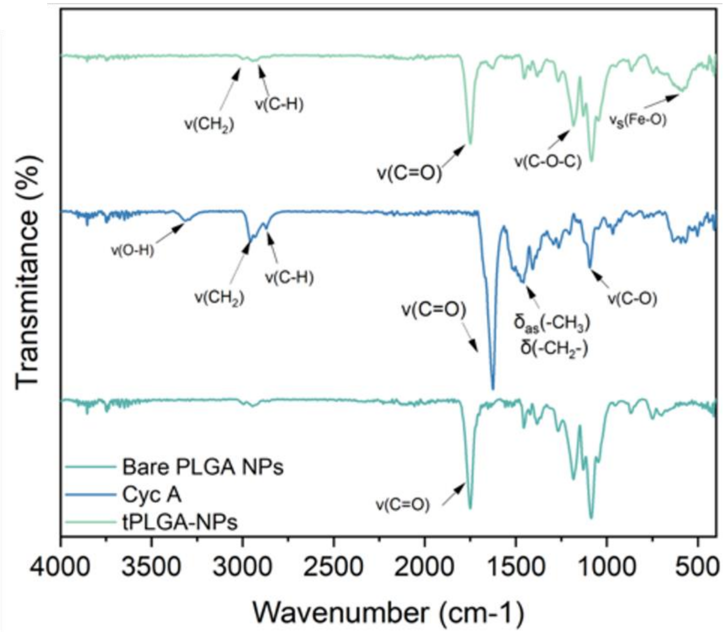

Figure S1 *– FTIR of tPLGA-NPs and controls.*

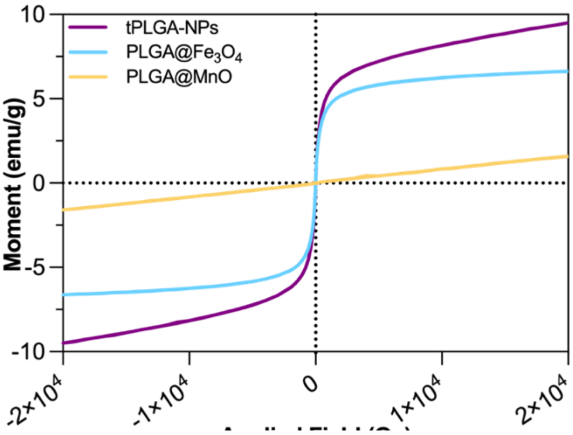

Figure S2 *- Magnetization curves as a function of applied field for tPLGA-NPs and controls.*

Flow cytometry

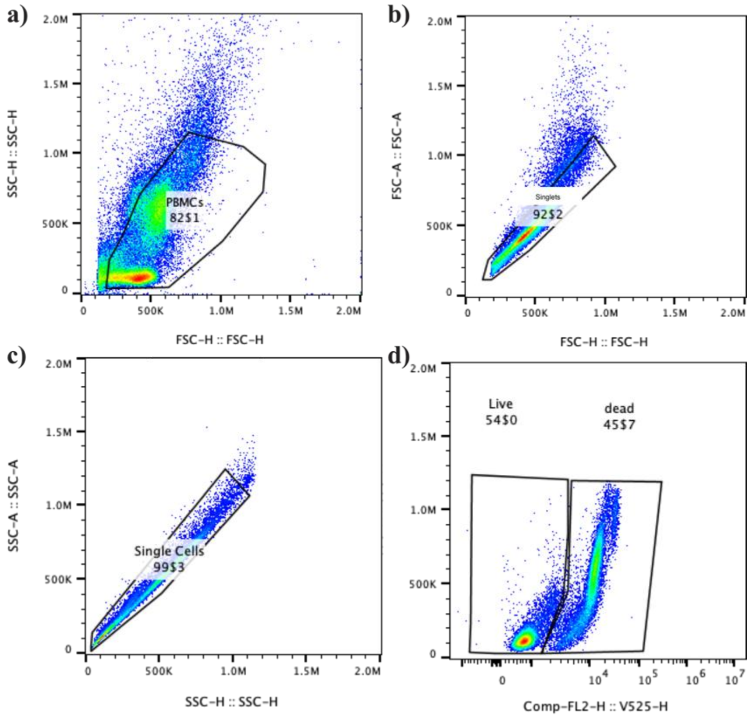

Figure S3 - *Flow cytometry gating strategy for live/dead cell analysis using Aqua dye. Healthy Donor PBMCs were first gated to exclude debris (a). Single cells were selected by removing doublets and aggregates (b, c). Live/dead discrimination was performed using Aqua viability dye, where live cells correspond to Aqua-negative and dead cells correspond to Aqua-positive (d).*

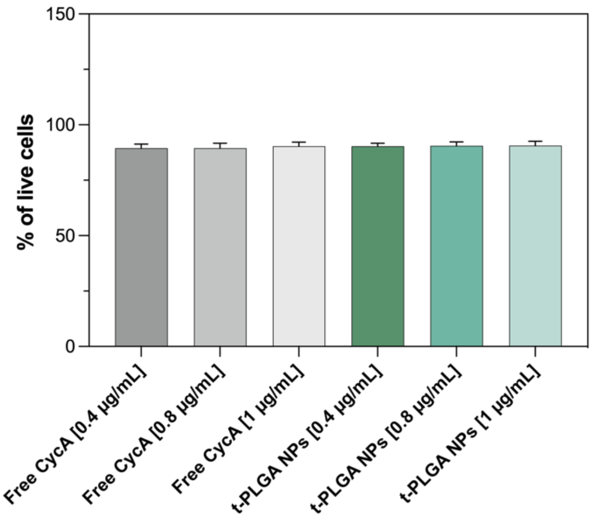

Figure S4 – *PBMCs cell viability determined by flow cytometry. The data represents the mean value and SEM of three independent donors.*
